# From Nature to Emergency: Cardiac Complications Following the Consumption of Unwashed Fruit

**DOI:** 10.7759/cureus.78455

**Published:** 2025-02-03

**Authors:** Mahmoud J. Tabouni, Anas AbuRamadan, Samah Awouda

**Affiliations:** 1 Internal Medicine, Hamad Medical Corporation, Doha, QAT; 2 Cardiology, Hamad Medical Corporation, Doha, QAT

**Keywords:** bradycardia, cardiotoxicity, pesticide toxicity, poisoning, sidr, ziziphus spina-christi

## Abstract

*Ziziphus spina-Christi* (Sidr) is widely used for its medicinal and nutritional properties, with documented pharmacological effects such as antidiabetic, sedative, and antihypertensive activities. However, its consumption, especially in unregulated settings, can pose significant health risks due to potential toxicity and contamination with pesticides. We report the case of a 25-year-old male who presented with severe abdominal pain radiating to the chest, profound bradycardia (heart rate in the 20s), hypotension (systolic blood pressure in the 50s), and autonomic symptoms after ingesting unwashed *Z. spina-Christi* fruit. Initial investigations revealed Mobitz type I second-degree atrioventricular (AV) block, transient ST depression, and elevated troponin levels, raising suspicion of acute coronary syndrome (ACS). However, comprehensive cardiac evaluations, including CT coronary angiography and cardiac MRI, excluded ischemic and structural cardiac causes. A detailed history revealed the ingestion of unwashed fruit, suggesting toxicity exacerbated by pesticide exposure. The patient responded well to supportive care, including atropine, and was discharged on the fourth day with a complete resolution of symptoms. This case highlights the diagnostic challenge posed by toxic ingestions mimicking ACS. Although it is not clear if *Z. spina-Christi* induced cardiotoxic effects at higher doses, the pesticide contamination could have also contributed to cardiotoxicity. Clinicians should consider toxicological etiologies in patients presenting with atypical gastrointestinal and cardiovascular symptoms, especially when linked to potential exposures. Early recognition and prompt management are critical for favorable outcomes.

## Introduction

The use of plants as food and remedies for various health conditions dates back to ancient times, yet only a limited number of these plants have been studied scientifically [1]. Although often perceived as less harmful than synthetic drugs, there is limited information on the toxicity of plants and herbal products [2]. This belief may be misguided, as many plants previously considered completely harmless have been scientifically proven to cause various adverse effects on living organisms [3]. *Ziziphus spina-Christi,* known as Sidr (Arabic), is thought to have originated from Sudan and grows in various regions throughout the world, especially the tropics [4]. Parts of *Z. spina-Christi* have been reported to have pharmacological effects such as antidiabetic, anti-nociceptive, central nervous system (CNS) modulating and antimicrobial activities [5]. The fruits of this plant have been reported for their antioxidant properties, while its seeds are recognized for their sedative effects [6, 7]. The flower, leaf and fruit of *Z. spina-Christi* are effective for controlling and treating hypertension [8]. Although this plant offers notable medical benefits, other studies emphasize the need for caution in its consumption due to potential adverse effects on vital organs caused by components like tannins, saponins, alkaloids, flavonoids, phenols and glycosides [9]. In this case report, we highlighted the potential cardiac effects of this fruit, exacerbated by the additional impact of pesticide exposure on the human body. After reviewing the literature, organophosphates were identified as the most commonly used pesticides in the region, and it is plausible that this type of pesticide contributed significantly to the patient’s clinical presentation [10]. Organophosphate exposure could have worsened the observed bradycardia, hypotension, and autonomic symptoms, amplifying the potentially suspected toxic effects of *Z. spina-Christi*. This observation underscores the importance of assessing both natural and environmental factors in toxicological cases.

## Case presentation

A 25-year-old male, a non-smoker with no significant medical or surgical history, presented to the Emergency Department (ED) at Heart Hospital in Doha, Qatar. The patient had an acute abdominal pain radiating to the chest, described as severe (9 out of 10 on a numerical rating scale) burning sensation. The patient sought help 20 minutes after the onset of pain, and emergency medical services (EMS) arrived 10 minutes later. The patient reported concurrent symptoms of sweating and shortness of breath followed by two episodes of vomiting, each consisting of a small amount of normal gastric contents, blurred vision and dizziness. The patient denied any fever, syncope, or loss of consciousness.

**Figure 1:**
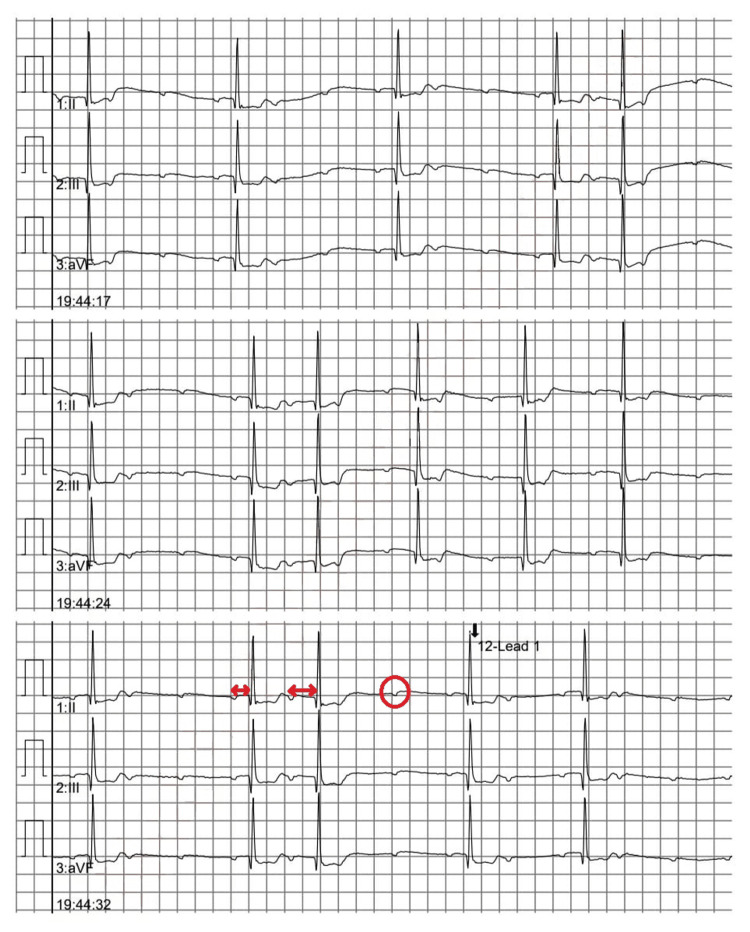
Mobitz type I Mobitz Type I (Wenckebach) second-degree atrioventricular (AV) block. This rhythm is characterized by a progressive prolongation of the PR interval (marked with arrows) until a QRS complex is dropped (indicated by circle). The ECG demonstrates this classic Wenckebach pattern in leads II, III, and arteriovenous fistula (aVF).

**Figure 2:**
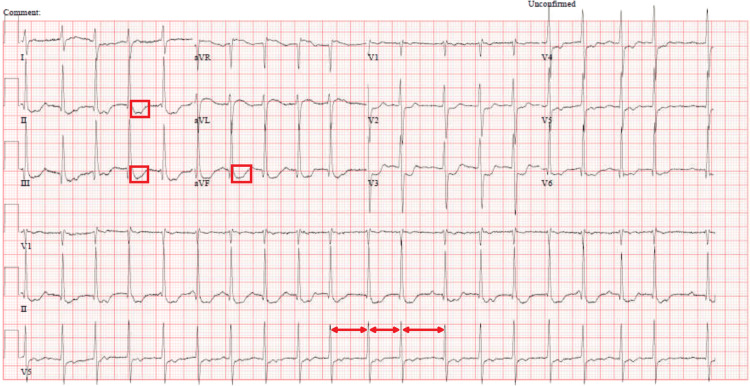
Atrial fibrillation with rapid ventricular response This ECG shows irregularly irregular heart rate (marked with arrows) with no P waves and a rapid ventricular response (heart rate 120); there is also a marked ST depression in leads II and III (indicated by squares). These changes strongly indicate myocardial ischemia.

**Figure 3:**
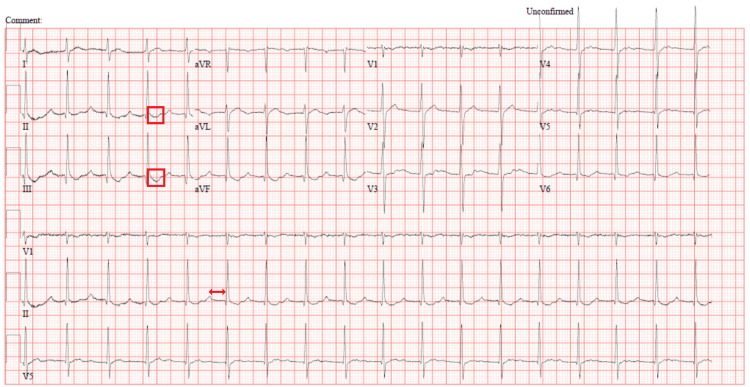
Tachycardia and first-degree atrioventricular block Sinus tachycardia (heart rate 105 beats per minute) with a first-degree atrioventricular (AV) block (indicated by arrow) and nonspecific ST and T wave abnormalities (highlighted with squares). The ECG findings correspond to transient electrical and repolarization disturbances seen in the patient during acute presentation.

Upon arrival of EMS, the patient was noted to be profoundly bradycardic, with a heart rate of 23 beats per minute (bpm), and hypotensive, with a systolic blood pressure of 56 mmHg. EMS initiated resuscitation, administering an epinephrine infusion 1 microgram per minute, ketamine 200 mg, and temporary transcutaneous pacing. Initial electrocardiogram (ECG) in the ED showed a Mobitz Type I second-degree atrioventricular (AV) block (Figure 1). Shortly (15 minutes) after receiving 1 mg of atropine in ED, the patient developed atrial fibrillation with rapid ventricular response, accompanied by transient, marked ST depression in leads II and III noticed on the monitor and confirmed with ECG (Figure 2). Then ECG was repeated after 10 minutes due to a change in rhythm on the monitor and showed sinus tachycardia at 105 bpm with a first-degree AV block and nonspecific ST and T wave abnormalities (ST depression and T wave inversion) (Figure 3). He then reported a significant reduction in chest pain after it had persisted for a total duration of three hours.

Upon further review of the patient’s history, he reported no known allergies, recent travel, or toxic exposures. His family history was notable for coronary artery disease in his father, diagnosed at the age of 57. Additionally, the patient’s body mass index (BMI) was 23, indicating a normal weight range.

On examination, the patient appeared drowsy, pupils were constricted (2-3 mm) but reactive to light, with otherwise unremarkable neurological, respiratory and cardiovascular findings, except for tachycardia. Other investigations included a transthoracic echocardiogram (Table 1, Video 1), which showed normal cardiac function. Laboratory results at admission are shown in Table 2. A urine drug screen was negative for amphetamines, cannabinoids, cocaine, opiates and phencyclidine. The patient was admitted for continuous cardiac monitoring, intravenous fluids (65 mL/hour and he received total 3 liters during his admission), and serial (every 6 hours then daily) troponin measurements to rule out acute coronary syndrome (ACS).

**Table 1:** Transthoracic Echocardiography IVS: Interventricular septum, MV: mitral valve, AV: aortic valve, TV: tricuspid valve, PV: pulmonic valve; LVEF: left ventricular ejection fraction, LV: left ventricle. Conclusion: Normal transthoracic echocardiographic study.

Findings:	
Left Ventricle	Normal size left ventricle. Normal global systolic LV function. Biplane LVEF is calculated at 60%. No regional wall motion abnormality. Normal diastolic LV function. Normal left ventricular wall thickness.
Right Ventricle	Normal size right ventricle. Normal RV function.
Left Atrium	The left atrium is normal in size.
Right Atrium	The right atrium is normal in size.
IVS	Normal thickness.
Mitral Valve	Normal morphology and function.
Aortic Valve	Normal morphology and function.
Tricuspid Valve	Normal morphology and function.
Pulmonic Valve	Normal morphology and function.
Aorta	Normal.
Pericardium	No pericardial effusion.

**Video 1:** Transthoracic Echocardiography RA: right atrium, LA: left atrium, RV: right ventricle, LV: left ventricle. This echocardiography four-chamber view reveals normal chamber sizes and normal global systolic left ventricular (LV) function, with no evidence of regional wall motion abnormalities.

**Table 2:** Laboratory Results at Admission Hs-TnT: High-sensitivity troponin-T, TSH: thyroid-stimulating hormone, Cr: creatinine, CK: creatine kinase, Mb: myoglobin, K: potassium

Parameter	Result (Admission)	Reference Range
Hs-TnT	22 ng/L	<14 ng/L
TSH	0.90 mIU/L	0.4–4.2 mIU/L
Lactic acid	4.3 mmol/L	0.5–2.2 mmol/L
Cr	116 μmol/L	44–106 μmol/L (male)
CK	180 U/L	25–200 U/L
Mb	134 ng/mL	28–72 ng/mL
K	5.6 mmol/L	3.5–5.1 mmol/L

**Figure 4:**
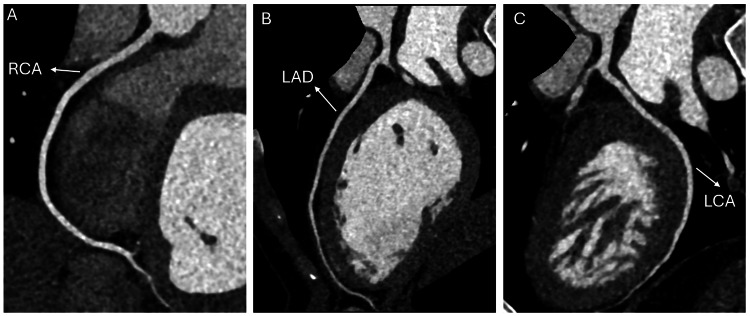
CT Cardiac angiogram coronary Normal origins and courses of right coronary artery (RCA) (A), left anterior descending artery (LAD) (B) and left circumflex artery (LCA) (C) with no stenosis or filling defects.

**Figure 5:**
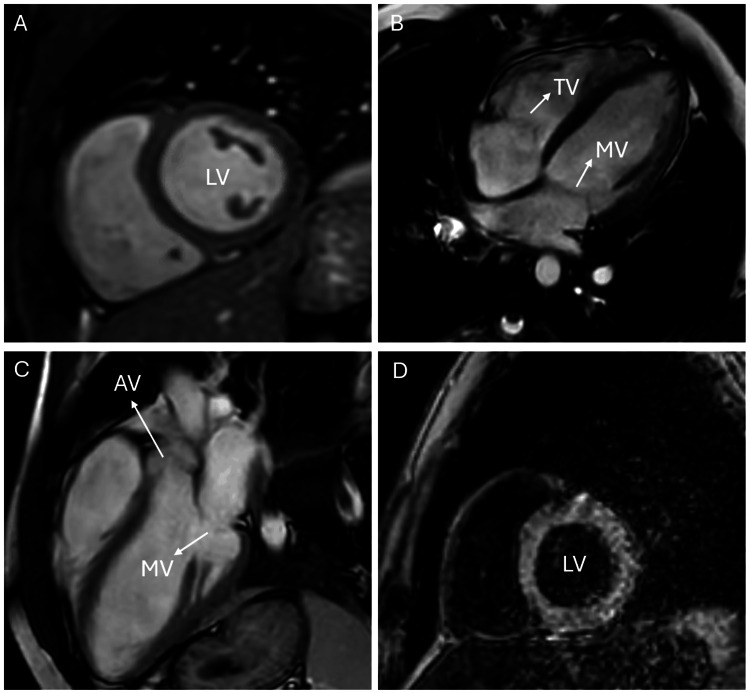
MRI Cardiac morphology function Multiple planes in cardiac MRI showing normal appearances. (A) The short-axis view shows no (LV) left ventricular wall abnormalities (B) The four-chamber view shows normal (MV) mitral and (TV) tricuspid valves (C) The left ventricular outflow tract view shows normal (AV) aortic and (MV) mitral valves. (D) The Post contrast short-axis view shows normal enhancement pattern.

**Figure 6:**
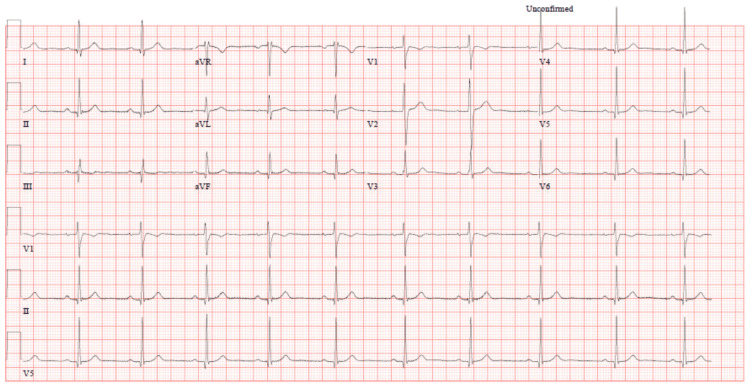
Normal ECG Normal ECG recorded on the third day of admission. No abnormalities in rhythm, conduction, or repolarization are observed, indicating resolution of the transient cardiac disturbances associated with intoxication.

Repeated lab tests (troponin after 6 hours, and other labs after 24 hours) showed a rising trend in troponin levels (initially 22 ng/L, peaking at 176 ng/L), alongside elevated lactic acid (3 mmol/L), creatinine (83 μmol/L), and potassium (5.1 mmol/L). These findings, coupled with ECG abnormalities, raised suspicion for acute coronary syndrome (ACS) and hypoperfusion, prompting further investigations. A CT coronary angiogram (Table 3, Figure 4) and 24-hour Holter monitoring (Table 4) revealed no abnormalities, and cardiac MRI (Table 5, Figure 5) excluded myocarditis or structural heart disease. By the third day, troponin levels had normalized, and ECG findings had resolved (Figure 6).

**Table 3:** CT cardiac angiogram coronary Ca score: Calcium score, LMA: left main artery, LAD: left anterior descending artery, LCX: left circumflex artery, RI: Ramus intermedius artery, RCA: right coronary artery. Conclusion: No evidence of significant coronary artery disease noted.

Findings	
Origin and course of the left and right coronary circulation	Normal.
Dominant artery	Right coronary artery.
Ca score	0.
LMA	Normal.
LAD	Normal, no evidence of stenotic lesion seen.
First diagonal branch	Normal.
LCX	Normal, no evidence of stenotic lesion seen.
RI	Normal, no evidence of stenotic lesion seen.
RCA	Normal, no evidence of stenotic lesion seen.

**Table 4:** Holter Monitoring Findings HR: Heart rate, AFIB: Atrial fibrillation, QRS complex: A waveform representing ventricular depolarization on an ECG, bpm: Beat per minute, R-R Interval: The time interval between two successive R-wave peaks in an ECG, N-N Delay: Delay between normal-to-normal heartbeats, Ectopy: Abnormal heartbeat originating from outside the normal conduction pathway, Bigeminal cycles: A pattern where every second heartbeat is abnormal. Conclusion: The Holter findings are largely unremarkable with no ventricular ectopy or significant arrhythmias. Mild supraventricular ectopy is benign. Bradycardia episodes observed during sleep, which are typically physiological in nature. No significant trends in heart rate variability or occurrences of transient arrhythmias were observed during daytime activities.

Parameter	Value	Normal Range	Additional Notes
Total QRS Complexes	78,945	-	-
Maximum Heart Rate (HR)	94 bpm	60–100 bpm	Average for 1 minute
Minimum Heart Rate (HR)	45 bpm	60–100 bpm	Average for 1 minute
Average Heart Rate (HR)	61 bpm	60–100 bpm	-
Ventricular Ectopic Beats	0 (0.00%)	0–50 beats/day	-
Supraventricular Ectopic Beats	73 (0.09%)	0–100 beats/day	-
Time Classified as Noise	0.00%	<5%	-
Beats in Tachycardia (>100 bpm)	28 (0.04%)	0–50 beats/day	-
Beats in Bradycardia (<60 bpm)	40,634 (51.47%)	0–50 beats/day	-
AFIB Episodes	0	0	No atrial fibrillation episodes detected
AFIB Burden	0.00 minutes (0.00%)	0 minutes	-
Max R-R Interval	1.64 seconds	<2 seconds	-
Supraventricular Ectopy	0.09%	0–0.1%	-
Ventricular Ectopy	0.00%	0–0.1%	-
Isolated Supraventricular Beats	38	0–50 beats/day	-
Supraventricular Couplets	1	0–10/day	-
Supraventricular Triplets	0	0–5/day	-
Supraventricular Bigeminal Cycles	2	0–5 cycles/day	-
Supraventricular Runs (≥4 beats)	6 runs totaling 33 beats	0–10 runs/day	0% of total beats
Longest Supraventricular Run	7 beats	<10 beats	74 bpm
Fastest Supraventricular Run	6 beats	<10 beats	77
Tachycardia Episodes (≥3 beats at ≥120 bpm)	0	0	No episodes detected
Tachycardia Episodes (≥4 beats at ≥80 bpm)	0	0	No episodes detected
Bradyarrhythmia Pauses (>2.00 seconds)	0	0	No pauses detected
N-N Delays (>140%)	64	<100 delays/day	-

**Table 5:** MRI Cardiac morphology function LV: Left ventricle, RV: Right ventricle, EF: Ejection fraction, LVEDD: Left ventricular end-diastolic diameter, RVEDD: Right ventricular end-diastolic diameter, LA: Left atrium, RA: Right atrium, T2WI: T2-weighted imaging, EDV: End-diastolic volume, ESV: End-systolic volume, SI: Stroke index. Conclusion: Normal biventricular volumes and function. No overt myocardial edema or fibrosis. No CMR evidence of myocarditis.

Parameter	Finding	Normal Range	Comments
LV			
LV (EF)	56%	57–77%	Slightly below normal range, not clinically significant
LVEDD	5.3 cm	≤5.6 cm	Normal
Wall Thickness (Max)	8 mm	≤10 mm	Normal
Regional Wall Motion	No abnormalities	No abnormalities	Normal
RV			
RV (EF)	50%	47–67%	Normal
RVEDD	4.2 cm	Normal for body size	Normal
LA			
LA Area	17.1 cm²	≤20 cm²	Normal
RA			
RA Area	20.4 cm²	≤22 cm²	Normal
Valves	No abnormalities	No abnormalities	Normal
Pericardium	Normal	No effusion	Normal
T2WI	No myocardial edema	None	Normal
Native T1 Mapping	959 ms	965–1055 ms	Slightly below normal, not clinically significant
Native T2 Mapping	44 ms	50–55 ms	Slightly below normal, not clinically significant
Post-Gadolinium Imaging			
Early Phase	No LV/RV thrombus	None	Normal
Late Phase	No myocardial enhancement	None	No infarction or overt fibrosis
Left Ventricle Indexed Quantitative Parameters			
LV (EDV)	104 ml/m²	68–112 ml/m²	Normal
LV (ESV)	46 ml/m²	16–44 ml/m²	Slightly above normal, not clinically significant
SI	58 ml/m²	44–76 ml/m²	Normal
Myocardial Mass (End-Diastole)	55 g/m²	47–87 g/m²	Normal
Right Ventricle Indexed Quantitative Parameters			
RV (EDV)	108 ml/m²	74–134 ml/m²	Normal
RV (ESV)	54 ml/m²	25–62 ml/m²	Normal
SI	54 ml/m²	41–77 ml/m²	Normal

**Figure 7:**
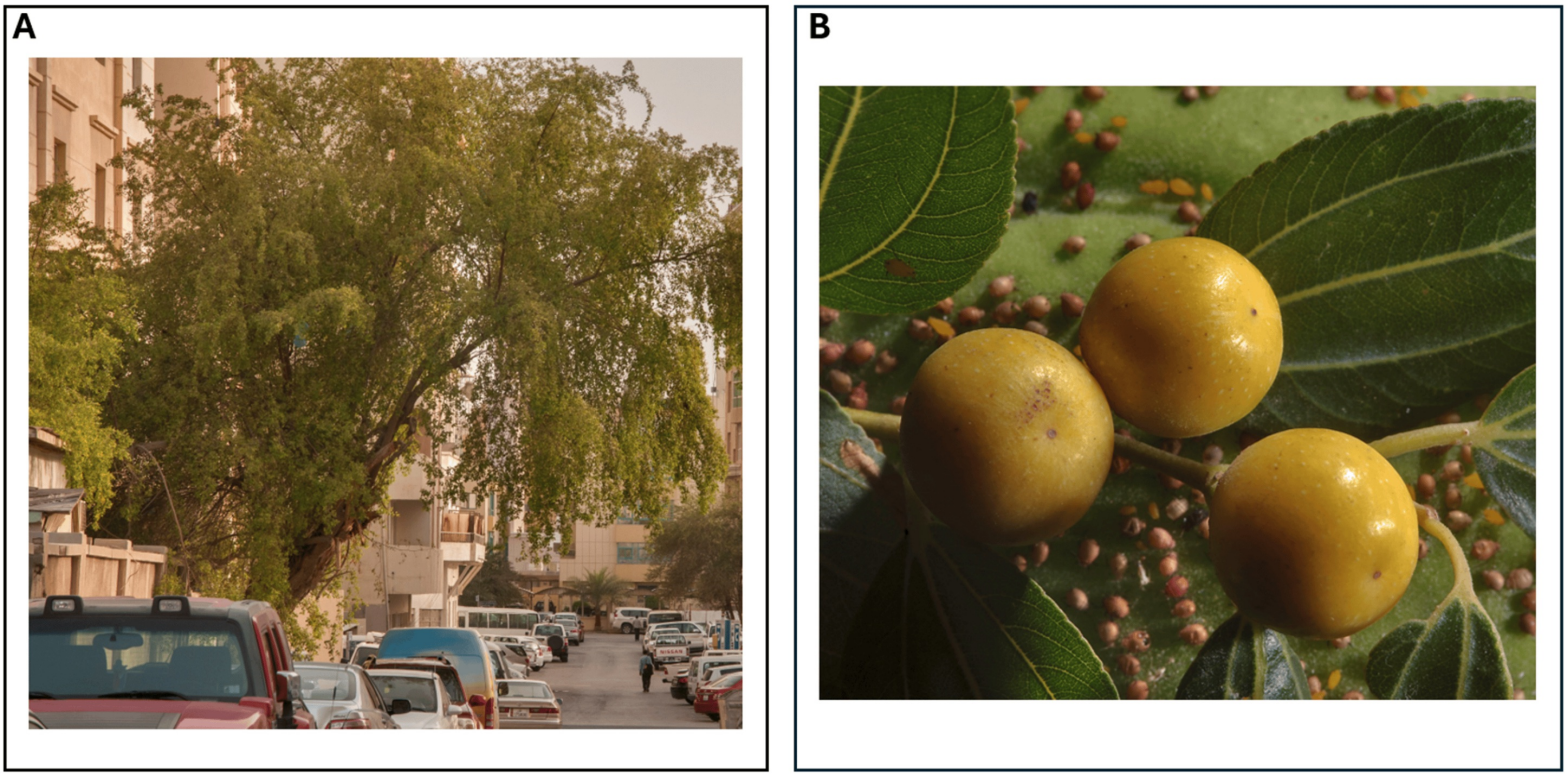
Sidr (Ziziphus spina-Christi) tree and Fruits (A) Large Sidr tree (*Ziziphus spina-Christi*) on Al Khattabi Street, Al Doha Al Jadeeda area, Doha, Qatar. (B) Fruits of Christ's thorn jujube (Sidr tree, *Ziziphus spina-Christi*) collected near Dareen Tower in West Bay, Doha, Qatar. Image source: Flora of Qatar. (n.d.). Ziziphus spina-Christi. Retrieved January 13, 2025, from https://www.floraofqatar.com/ziziphus_spina-christi.htm. Used with permission from the website administrator Alexey Sergeev.

During further history-taking, the patient and his friend disclosed that he had ingested multiple unwashed Sidr (*Z. spina-Christi*) fruits (Figure 7) from a street tree 1-2 hours before symptom onset. This history, combined with the patient’s bradycardia, hypotension, abdominal and chest pain, and significant improvement with supportive measures, strongly suggests poisoning from a *Z. spina-Christi*-derived substance, potentially exacerbated by pesticide exposure. 

The toxicology team reviewed the case and concluded that the diagnosis could be confidently established based on the exposure history, clinical findings and ruling out other causes like structural abnormality, ischemic heart disease, arrhythmogenic syndromes or myocarditis through unremarkable CT angiogram, Holter monitoring and cardiac MRI findings support the diagnosis of transient, reversible etiology like plant toxicity. They did not recommend further diagnostic tests, given the patient’s marked improvement and the lack of specific confirmatory tests for *Z. spina-Christi* toxicity or organophosphate toxicity (red blood cell acetylcholinesterase activity test is not available in our facility).

The patient’s condition improved, and he was discharged home on the fourth day of hospitalization with normal ECG and troponin levels, and no residual symptoms. We advised him to avoid any future ingestion of unwashed or unfamiliar street fruit, and a follow-up appointment was scheduled for the patient; however, unfortunately, he did not turn up for the appointment. This case underscores the pivotal role of detailed history-taking in identifying toxic ingestions. Poisoning should be considered in cases presenting with cardiovascular and gastrointestinal symptoms resembling acute coronary syndrome, especially when there is a history of possible exposure. Prompt recognition and treatment can lead to significant clinical improvement and saving life.

## Discussion

This case highlights the diagnostic challenges in a young, otherwise healthy patient with a combination of gastrointestinal, cardiac, and autonomic symptoms. Initially, the focus was on ACS due to elevated troponin levels and ECG abnormalities, a common diagnostic approach in emergency settings [11]. However, further investigation revealed an atypical toxicological etiology.

The patient presented with severe bradycardia (heart rate in the 20s) and hypotension (systolic blood pressure in the 50s), prompting urgent interventions, including adrenaline infusion, transcutaneous pacing, and atropine administration for stabilization [12]. Such profound bradycardia typically suggests serious underlying conditions, such as myocarditis, cardiovascular pathology, or the effects of pharmacologic agents [13].

ECG findings showed Mobitz Type I second-degree AV block, which later evolved into atrial fibrillation with transient ST depression in the inferior leads, can be related to the treatment that patient received, especially atropine [14]. Elevated troponin-T levels (initially 22 ng/L, rising to 176 ng/L) suggested myocardial injury; as a result, ACS was initially suspected. However, both coronary CT angiography and 24-hour Holter monitoring were unremarkable, effectively ruling out ischemic causes and persistent arrhythmias. Additionally, transthoracic echocardiography and cardiac MRI revealed no myocarditis, stress-induced cardiomyopathy (Takotsubo cardiomyopathy) or structural abnormalities, leading clinicians to consideration of alternative explanations [12, 13, 15].

A critical clue emerged when the patient reported ingesting unwashed Sidr (*Z. spina-Christi*) from a street tree two hours prior to symptom onset. This history, combined with the signs and symptoms of bradycardia, hypotension, abdominal pain, miosis, a positive response to supportive treatment, and the rapid resolution of symptoms, strongly suggested toxicity as a likely cause. By reviewing the literature, we found that *Z. spina-Christi* fruit is well established to be toxic at higher doses, causing significant alterations in liver and renal function tests, as well as histological changes in the liver [16, 17]. Research conducted on frogs has shown that cardiac effects due to *Z. spina-Christi* can range from bradycardia to cardiac arrest, depending on the ingested dose of the plant [18]. The ingestion of unwashed fruit raises the possibility of pesticide exposure as a contributing factor that may have amplified the patient's clinical presentation including the elevated troponin [19]. Pesticide toxicity, including organophosphate poisoning, is unlikely to be the sole cause of this patient's presentation, although some cholinergic effects such as bradycardia, abdominal pain and miosis were observed. Key features of severe organophosphate toxicity - such as unresponsiveness, muscle fasciculations, diarrhea, excessive salivation, lacrimation, and urinary incontinence - were absent, and the patient's heart rate improved significantly with a single dose of atropine, while severe organophosphate poisoning typically requires pralidoxime and multiple doses of atropine for stabilization [20].

## Conclusions

This case highlights the importance of detailed history-taking and a high index of suspicion when evaluating patients presenting with a combination of cardiovascular, gastrointestinal, and autonomic symptoms. The ingestion of unwashed *Z. spina-Christi* fruit, likely contaminated with pesticides, was the probable cause of the patient's profound bradycardia, hypotension, and elevated troponin levels, which initially mimicked acute coronary syndrome. The patient's symptoms resolved with supportive care, including atropine administration and cardiac monitoring, emphasizing the importance of prompt recognition and management of plant poisoning. 

The future implications of this study are as follows: (1) Further research is needed to identify the specific compounds in *Z. spina-Christi* responsible for its toxic effects on the heart and to understand the mechanisms of action, particularly in combination with contaminants like pesticides. (2) Educational campaigns should emphasize proper cleaning techniques for fruits and vegetables, such as rinsing under running water, scrubbing tough skins, and soaking delicate produce. These efforts can reduce the risk of toxin or pesticide exposure. (3) Stronger policies on pesticide regulation, residue testing, and farmer education are critical to minimize contamination risks. Authorities should monitor pesticide use and promote safer agricultural practices. (4) Hospitals should equip themselves with tools like plant toxin assays and red blood cell cholinesterase testing to better diagnose and manage plant and pesticide-related toxicities.
